# Taking a Comparative Approach: Analysing Personality as a Multivariate Behavioural Response across Species

**DOI:** 10.1371/journal.pone.0042440

**Published:** 2012-07-31

**Authors:** Alecia J. Carter, William E. Feeney

**Affiliations:** 1 Fenner School of Environment and Society, Australian National University, Canberra, Australian Capital Territory, Australia; 2 School of Marine and Tropical Biology, James Cook University, Townsville, Queensland, Australia; 3 Research School of Biology, Australian National University, Canberra, ACT, 0200, Australia; Liverpool John Moores University, United Kingdom

## Abstract

Animal personality, repeatable behaviour through time and across contexts, is ecologically and evolutionarily important as it can account for the exhibition of sub-optimal behaviours. Interspecific comparisons have been suggested as important for understanding the evolution of animal personality; however, these are seldom accomplished due, in part, to the lack of statistical tools for quantifying differences and similarities in behaviour between groups of individuals. We used nine species of closely-related coral reef fishes to investigate the usefulness of ecological community analyses for the analysis of between-species behavioural differences and behavioural heterogeneity. We first documented behavioural carryover across species by observing the fishes' behaviour and measuring their response to a threatening stimulus to quantify boldness. Bold fish spent more time away from the reef and fed more than shy fish. We then used ecological community analysis tools (canonical variate analysis, multi-response permutation procedure, and permutational analysis of multivariate dispersion) and identified four ‘clusters’ of behaviourally similar fishes, and found that the species differ in the behavioural variation expressed; some species are more behaviourally heterogeneous than others. We found that ecological community analysis tools are easily and fruitfully applied to comparative studies of personality and encourage their use by future studies.

## Introduction

Selection should theoretically favour individuals that adapt their behaviour to their current ecological and social circumstances, however this is not always observed in nature [1]. Animal personality theory can explain these observations and suggests behaviour is more ‘constrained’ to certain behavioural types [1], [2]. While the definition of animal personality can vary between studies [2], [3], it is agreed that animal personality refers to low within-individual variation in behaviour relative to between-individual variation in behaviour; behaviour has to be repeatable through time to be considered a personality trait [2], [4], [5], [6], [7]. By being able to explain the existence and exhibition of both optimal and suboptimal behaviours, animal personality has been recognised as an ecologically and evolutionarily important theory [1], [2], [6], [8].

A fundamental question within behavioural ecology concerns the evolutionary basis for the existence of animal personalities [2]. Recent conceptual work has suggested that intraspecific population and/or interspecific comparisons could be used to investigate these types of questions [9], [10], [11]. For example, intraspecific population comparisons have identified predation as a driving force behind the maintenance of correlated behavioural traits [12], [13]. However, studies of interspecific differences in personality traits have produced mixed results. For example, Mettke-Hofmann et al. [11] successfully related differences in exploration and neophobia in 61 species of parrots (Psittacidae) to variation in diet and ecology. Webster et al. [10], however, found that three- and nine-spined sticklebacks (*Gasterosteus aculeatus* and *Pungitius pungitius*, respectively) differed significantly in the time spent in open water, but there was no difference in activity or latency to attack prey (boldness) between the species. Further, Mettke-Hofmann et al. [9] showed that resident and migratory warbler species (*Sylvia melanocephala momus* and *S. borin*, respectively) exhibited differences in the repeatability of and correlations between exploration and boldness. Thus, while the importance of comparative studies to animal personality research is recognised [2], few studies have tested these ideas, and few traits have been investigated.

Comparative approaches towards animal personality face two primary problems: 1) finding a behavioural assay that is comparable between species [2] and 2) identifying similarities and differences between groups of individuals, especially for multivariate responses such as behaviour. Finding a test that is comparable between species can be somewhat overcome by focusing on groups of closely related species. However, techniques to identify similarities and differences in behaviour are less established in behavioural ecology. We suggest that descriptive and statistical techniques used in multivariate ecological community analysis could be useful when studying interspecies differences in behaviour. For example, canonical variate analysis (CVA) is a data reduction technique similar to principal components analyses (PCA) that further uses a categorical grouping factor to separate groups [14]. Previously, it has been used to distinguish species based on morphological measurements [14], [15], [16]. Further, community composition analyses allow grouping of, in this case, species into similar and dissimilar ‘clusters’ by identifying compositional dissimilarity and heterogeneity. By identifying behaviourally similar and distinct species, and behaviourally homogeneous and heterogeneous species, we suggest that these statistical tools may provide a base to investigate the factors driving between-species differences in behaviour.

Here we apply ecological community analysis tools to analyse multivariate behaviour in nine species of predator-naïve recruit coral reef fishes (Pomacentridae). We chose this group of species (see Methods) because they are closely related to one another, often occur in sympatry but comprise different ecological niches, and are locally abundant. Further, life-history bottlenecks, such as that found following settlement in coral reef fishes, are ideal systems in which to investigate the evolutionary basis of animal personality as selection (primarily predation) is very high [17], [18], [19]. Accordingly, recent interest has been placed on the importance of personality for survival in these systems, and traits that have received attention (primarily boldness) have been shown to be repeatable through time [20], [21], [22], [23], [24]. In this study we first investigate the ecological relevance of boldness behaviour across species. Second, we use descriptive techniques to identify whether there are differences between species' behaviour, and finally we identify differences in behavioural dissimilarity and heterogeneity between species using community analysis tools ‘borrowed’ from the ecological literature.

## Methods

### Ethics statement

All research was conducted in accordance with the James Cook University Animal Ethics guidelines (Permit Number: A1520).

### Study area and species

We studied nine species of pre-settlement juvenile damselfishes (Pomacentridae: *Chrysiptera rollandi* (*n* = 12), *Dischistodus perspicillatus* (*n* = 10), *Neopomacentrus azysron* (*n* = 10), *Pomacentrus amboinensis* (*n* = 10), *P. chrysurus* (*n* = 12), *P. coelestis* (*n* = 15), *P. moluccensis* (*n* = 13), *P. nagasakiensis* (*n* = 10), *P. wardi* (*n* = 12)) on the shallow reef flats surrounding Lizard Island (14°40′S, 145°27′E), Northern Great Barrier Reef, Australia in November and December 2009. Like most species of coral reef fishes, these species have a dispersive/pelagic larval stage followed by recruitment to the reefs for a more benthic juvenile/adult stage [25]. We caught all fishes during the recruitment process but prior to settlement to the reef using light traps [20], [21], [22], [26], [27]. This collecting technique minimizes phenotypic bias of the experimental population, as at recruitment the population is drastically augmented, most notably by extremely high rates of predation which can result in over 60% mortality of a cohort within 48 h of settlement [17], [19], [28]. Further, mixing of populations during the pelagic stage [29] and the high number of individual recruits helps ensure independence of replicates. Following capture, the fishes were transported to the research station at Lizard Island where they were sorted by species using a hand-net [20], [21], [22] and kept overnight in flow-through aquaria to recover prior to experimentation.

### Experimental design

Nine patch reefs were created out of coral fragments and coral rubble on the shallow (<5 m) sand banks surrounding Lizard Island. Patch reefs were constructed approximately 10 m apart and approximately 10 m from the reef edge [20], [21], [22]. Water temperatures during this time varied between 26.2°C to 29.5°C.

### Behavioural assays

Experimental fish were randomly selected from the previous day's catch [20], [21], [22]. Experimental fish were transported to the field and one fish was placed on each patch reef and allowed 30 min to acclimate before behavioural observations. Following acclimation, each individual fish was observed for a 3 min period by one observer (WEF) using self contained breathing apparatus (SCUBA) to obtain behavioural assays and a commonly used boldness measure [20], [21], [22]. Observed behaviours during assays comprised: total distance moved during the 3 min observation period, percentage of time spent 0, 2, 5 and 10 cm from the patch reef, proportion of time spent feeding (actively striking), lurking (not feeding) and observing (actively looking around environment while slowly rotating), furthest distance travelled from the patch reef, and proportion of time spent at the bottom third, middle third and top third (including above) of the patch reef. We measured the furthest distance travelled from the reef and proportion of time at different heights on the reef as the predatory assemblage differs at different parts of the reef [30], [31], which may have implications for fish behaviour. In order to assess whether the recorded behaviours, which were used to assess species' differences in behaviour, were ecologically relevant for animal personality studies, we further recorded strike rate (a measure of feeding success) and individual boldness (using a standard boldness assay) [21].

We defined boldness as the response to risk [13]. Boldness was measured at the end of the 3 min observation periods using a startle response: the observer extended a pencil toward the fish until the fish responded (see below) to its approach [21]. Boldness was recorded on a four-point scale from 0–3 in increments of 1. A boldness measure of zero was defined as the fish hiding in the coral and seldom emerging following the approach of the pencil; 1 was retreating in response to the pencil and not emerging for more than 5 seconds; 2 was retreating to the coral upon approach of the pencil but quickly emerging (<5 sec) and resuming normal behaviours (e.g. feeding); and 3 was readily venturing away from the patch reef, dodging the pencil without returning to the patch and continuing normal behaviours.

### Statistical methods

#### Describing a behavioural syndrome

To verify that the recorded behaviours were important in personality studies (i.e. that they correlated with an experimental measure of boldness), we used a ‘traditional’ approach (principal component analysis, PCA) to reduce the 12 recorded behaviours to fewer variables to investigate the effect of boldness on these traits. PCAs are commonly used in studies of animal personality [12], [32] as a data reduction technique, however we note that this method may potentially violate the assumption of independent samples due to replication within species (if there were indeed between-species differences in behaviour). We use it here to demonstrate the relationship between behaviour, boldness and foraging success, assuming independence of observations. Maximum distance travelled and distance moved in 3 min were log transformed to satisfy assumptions of normality and variables recorded as a proportion were ranked in order to break the bounds of the proportions. All data were then mean-centred and standardised for analysis in the PCA. We used Horn's parallel analysis [33] to determine the number of components to be retained for further analysis.

We next used linear mixed effects (LME) models in order to investigate the relationship between PC1 (boldness-activity behaviour), PC2 (time budgets), PC3 (time spent observing at 0 cm from the reef) and PC4 (time spent in the middle of the reef) and individual boldness measured using a traditional experimental procedure (startle response) and feeding success (strike rate). Species was included as a random effect in all the models. Strike rate was log-transformed in order to satisfy assumptions of normality. We performed three LME models to investigate the relationship between a) boldness score as a response and PCs 1–4 as predictors, b) strike rate as a response and PCs 1–4 as predictors and c) boldness score as a response and strike rate as a predictor. We sequentially dropped non-significant (*t*<2) terms for a) and b) above until the minimal model remained. Residuals of the models were checked for normality. We further investigated these relationships within each species by running models a–c above using linear models (models a, c) or a generalized linear model with a log link (model b). However, we investigated only the effects of PCs 1 and 2, and did not included PCs 3 and 4 (as they had no effect on boldness or strike rate, see Results below), to avoid overparameterisation.

#### Applying ecological community analysis tools

In order to assess the application of ecological community tools to comparative personality studies, we first investigated the recorded behaviours using multivariate descriptive techniques. We used canonical variates analysis (CVA) to group species according to behaviour [15]. CVA first analyses a matrix of variable values in much the same way as PCA, however CVA maximises the variance explained between groups while PCA maximises the variation among individuals (for a comparison of the results of these methods, see Supplementary Fig. S1). It then calculates the group's means, in this case species' centroids (the geometrical mean), and plots the first two components as a bivariate plot. Like PCAs, CVAs are descriptive techniques that can be used for data reduction, and are interpreted in a similar fashion. However, unlike PCAs, variables are more or less represented by each CV on a scale of 0 (poorly represented) to 1 (well represented) rather than ‘loading’ on components. Well-represented variables suggest differences between groups in those variables. The ‘directions’ of each variable (whether ascending or decending, represented as axes on the plot) can be determined by the biplot: the labels are associated with the positive end of the variable. Axes that are closer together are more correlated.

Next, we calculated behavioural dissimilarity between species as a measure of behavioural distinctiveness (how different are species behaviourally from one another?) and behavioural heterogeneity (how variable are species behaviourally from one another?). We calculated behavioural distinctiveness using multi-response permutation procedure (MRPP) [34]. MRPP is a non-parametric multivariate statistical technique used to calculate whether there is a difference between *a prioi* defined groups of entities, in our case, species. It is a quantitative statistic, robust to unequal variance, non-normally distributed data and unbalanced designs through use of permutation between groups. Groups that are clustered in multidimensional space have lower average distances to their group centroid than their inter-group centroids; they are dissimilar to the other species. The average distance, weighted by group size, is referred to as a lower case delta, δ. Thus, if species' points are distinct from other species, the intraspecies average distances will be small compared to interspecies average distances. To test this, the data are permuted in MRPP; in each permutation, group ‘membership’ is randomised and the resulting δ values (δ_exp_) are compared to the observed δ (δ_obs_) to calculate a *p*-value. Finally, within-group agreement, *A*, is calculated as 1-(δ_obs_)/(δ_exp_) as a measure of how well the individuals ‘fit’ within their groups, in this case, species. That is, within-group agreement would be 1 if all individuals within each species behaved identically to one another, and 0 if all individuals behaved according to chance. Community ecology values of *A* are commonly *A*≈0.1, while values >0.3 are high [35]. We used Bray-Curtis distances on the untransformed (count) data in the package “vegan” with 1000 permutations [36]. Finally, we tested for differences in behavioural heterogeneity using permutational analysis of multivariate dispersion in the program PERMDISP2 [37], [38] with 999 permutations. Similar to MRPP, PERMDISP2 calculates within-group average distances to the group centroid and compares these values to other group's average distances. Thus, species with higher average distances to the group centroid relative to other species have higher behavioural heterogeneity than species with small average distances.

## Results

### Describing a behavioural syndrome

Fishes' average scores for each of the behaviours are listed in Table 1. We used Horn's parallel analysis to determine principal components (PCs) 1–4 be retained for further analysis. PCs 1–4 explained 39.1, 16.7, 12.4 and 10.2% of the variation, respectively (78.3% of the total variation). Proportion of time spent in the bottom third of the reef loaded positively on PC1, while proportion of time spent within 5 and 10 cm of the reef, in the top third of the reef and total and maximum distances moved loaded negatively on PC1 (Table 2). This suggests that lower values of PC1 indicate individuals that spent more time in ‘riskier’ areas (far from and high on the patch reef) and more time moving, and higher values indicate individuals that spent time in ‘safer’ areas (close to the bottom of the patch reef). PC1 is thus suggestive of a boldness-activity personality trait. PC2 explained conflicts in activity budgets, with low values indicating individuals that spent more time feeding and high values indicating individuals that spent more time lurking. Time spent at 0 cm from the reef and time spent observing loaded positively on PC3 while time spent at 2 cm from the reef and time spent lurking loaded negatively. This partly reflects the definitions of the activity budget behaviours and is again representative of a conflict in these budgets. Finally, PC4 explained time spent in the middle third of the patch reef, with high values indicative of individuals spending more time in the middle.

**Table 1:** Summary statistics (mean ± s.e.) for the behaviours collected during focal observations.

				Proportion of time spent (from reef)				Proportion of time spent at (of reef)			Proportion of time spent				
Species	*N*	Maximum distance travelled from reef	Total distance moved in 3 min	0 cm	2 cm	5 cm	10 cm	Bottom third	Middle third	Top third	Feeding	Lurking	Observing	Strike rate	Boldness
*C. rollandi*	12	5.33±1.97	12.91±7.82	1.67±3.26	51.67±36.39	42.50±34.08	4.17±11.65	60.42±45.15	26.25±29.70	13.33±21.98	84.58±11.95	12.50±10.55	0.83±2.89	107.00±23.36	2.58±0.51
*D. perspicillatus*	10	5.50±2.27	12.40±8.80	14.50±28.13	56.50±36.75	28.00±34.17	1.00±3.16	72.00±40.77	27.00±39.17	1.00±3.16	78.50±21.35	21.50±21.35	1.00±3.16	53.60±12.10	1.90±0.88
*N. azysron*	10	32.70±13.44	73.00±37.51	0.00±0.00	3.00±9.49	20.50±23.15	76.50±29.82	0.00±0.00	11.50±20.00	88.50±20.00	72.00±13.98	28.00±13.98	0.00±0.00	67.40±9.48	2.60±0.70
*P. amboinensis*	10	6.80±2.35	22.70±12.00	13.00±3.16	61.00±18.38	24.50±24.56	1.50±3.37	25.50±29.86	63.00±26.58	11.50±11.32	66.00±17.13	30.00±11.55	4.00±12.65	69.40±20.44	2.00±0.15
*P. chrysurus*	12	5.50±4.44	11.92±5.80	58.33±36.57	33.33±29.95	7.50±15.45	0.83±1.95	94.58±8.91	5.41±8.91	0.00±0.00	50.83±17.30	11.67±8.35	37.50±19.13	43.42±14.57	0.33±0.65
*P. coelestis*	15	15.80±18.34	52.33±46.50	8.67±15.52	49.33±30.52	28.67±20.04	13.33±23.50	32.67±42.34	43.33±36.04	24.00±32.41	84.33±18.60	14.33±15.68	1.33±5.16	125.07±38.26	2.60±0.63
*P. moluccensis*	13	8.00±4.98	23.85±17.76	15.38±3.16	54.23±28.27	27.31±26.66	3.08±5.22	6.54±22.12	49.23±37.52	44.23±38.51	70.00±25.17	20.77±19.35	9.23±18.01	53.85±22.21	2.38±0.65
*P. nagasakiensis*	10	6.70±2.50	27.50±17.99	8.00±8.23	73.50±10.01	17.00±11.35	1.50±3.38	10.00±13.94	58.50±30.37	58.50±30.37	73.00±18.89	16.00±9.37	5.00±7.45	84.50±10.55	2.50±0.71
*P. wardi*	12	9.33±12.23	36.50±44.80	9.17±2.24	73.33±34.07	7.50±11.18	10.00±23.35	85.42±26.92	10.42±19.82	4.17±7.93	60.00±28.60	38.33±26.57	1.67±5.77	64.41±31.42	1.67±0.89

**Table 2:** Behaviours measured during focal watches that were entered into a principal component analysis (PCA) and canonical variate analysis (CVA) and their loadings on PCs 1–4 and representation by CVs 1 and 2, respectively.

Behaviour	PC1	PC2	PC3	PC4	CV1	CV2
Maximum distance travelled	**−0.37**	0.14	0.00	0.00	**0.73**	0.09
Distance moved in 3 min	**−0.40**	0.15	0.00	−0.18	0.49	0.18
Proportion of time spent within 0 cm of the reef	0.25	0.21	**0.42**	0.24	0.28	0.53
Proportion of time spent within 2 cm of the reef	0.23	−0.18	**−0.53**	0.00	0.59	0.08
Proportion of time spent within 5 cm of the reef	−0.30	−0.27	0.00	**0.31**	0.03	0.14
Proportion of time spent within 10 cm of the reef	**−0.37**	0.21	0.15	−0.28	**0.87**	0.03
Proportion of time spent in the bottom third of the reef	**0.34**	−0.13	0.15	−0.25	**0.68**	0.01
Proportion of time spent in the middle third of the reef	−0.18	0.00	0.00	**0.78**	0.01	0.01
Proportion of time spent in the top third of the reef	**−0.36**	0.20	−0.11	0.00	**0.71**	0.03
Time spent feeding	−0.17	**−0.60**	0.12	−0.15	0.01	0.30
Time spent lurking	0.00	**0.51**	**−0.48**	0.00	0.01	0.32
Time spent observing	0.22	0.30	**0.47**	0.18	0.05	**0.89**
Variation explained (PCA)	39%	17%	12%	10%		
Adjusted eigenvalue (PCA)	4.10	1.58	1.19	1.03		

Bold type indicates behaviours that load on each principal component and behaviours that are well represented by the canonical variates. Note that the canonical variates give no indication of the direction of the relationships. The percentage of variation explained and the adjusted eigenvalues for each principal component are listed.

We investigated the ecological relevance of an experimental boldness assay on naturally occurring behaviour. The minimal models investigating the effects of PCs 1–4 on the experimental measure of boldness (the startle response) and strike rate included PCs 1 and 2 as explanatory variables in both models (Table 3). Across species, fish that spent more time active, feeding and in riskier areas of the patch reef had higher boldness scores (startle responses) and higher strike rates. Secondly, bolder individuals gained a potential fitness benefit through increased feeding rates (Table 3), although this is unsurprising given the above correlations. Finally, we investigated these relationships at the species level (see Supplementary Table S1), but found variable correlations between the recorded behaviours between species.

**Table 3:** The estimates of the variables in the minimal models (linear mixed effects models) investigating the relationships between the startle response and natural behaviour, strike rate and natural behaviour, and the startle response and strike rate.

Response	Predictor	β ± s.e.	N	df	*t*
Startle response	Intercept	2.07±0.16	109	92	12.51
	PC1 (Boldness-Activity)	−0.18±0.04			−5.00
	PC2 (Feeding/Lurking)	−0.11±0.05			−2.18
Strike rate	Intercept	66.68±4.06	109	92	3.04
	PC1 (Boldness-Activity)	−1.08±1.01			−5.89
	PC2 (Feeding/Lurking)	−1.13±1.02			−6.78
Startle response	Intercept	1.48±0.29	109	92	5.07
	Feeding rate	0.01±0.003			2.90

### Applying ecological community analysis tools

**Figure 1:**
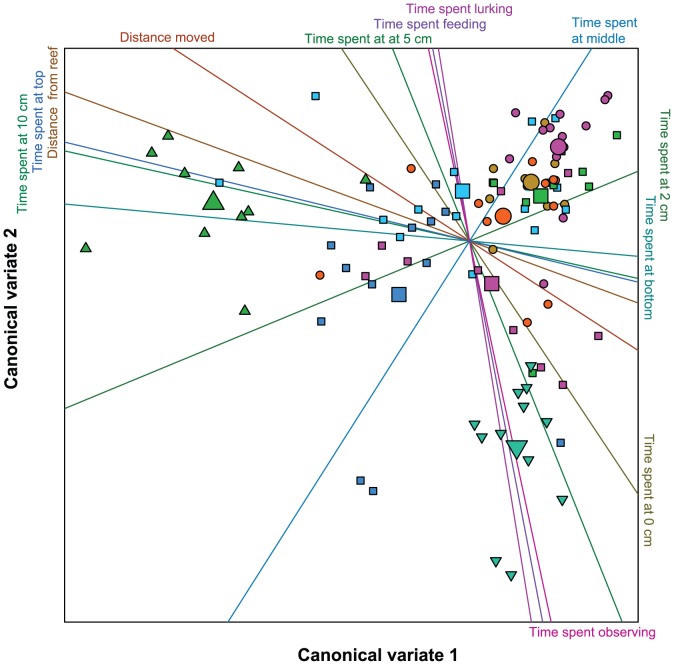
Position of individual fish and group centroids of fishes in multivariate behavioural space. Individual fish plotted on canonical variates 1 and 2. Coloured lines on the plot represent behavioural variables with labels attached to the positive end of each variable. Each smaller point represents an individual fish, with larger points indicating the centroids for each species. Each symbol represents fish that were classified *a posteriori* to belong to a group of similarly behaving fish (see Fig. 2); each colour represents a different fish species: *Chrysiptera rollandi* (orange circle), *Dischistodus perspicillatus* (brown circle), *Neopomacentrus azysron* (green upward pointing triangle), *Pomacentrus amboinensis* (light green square), *P. chrysurus* (aqua downward pointing triangle), *P. coelestis* (light blue square), *P. moluccensis* (blue square), *P. nagasakiensis* (purple square), *P. wardi* (pink circle).

We used CVA to group species according to the behaviour of the individuals. CVA revealed three distinct groups of species that differed in their behaviours on canonical variates 1 and 2 (Fig. 1) and thus warranted further investigation. Behaviours that were well represented by CV1 included the maximum distance travelled, the proportion of time spent 10 cm from the reef, in the top third of the reef and, in the opposite direction, in the bottom third of the reef (Table 3, Fig. 1). Time spent observing was well represented by CV2 (Table 3, Fig. 1). That is, species of fish differed most in the ‘risky’ parts of the reef they used (CV1) and the behavioural state they were in (CV2).

**Figure 2:**
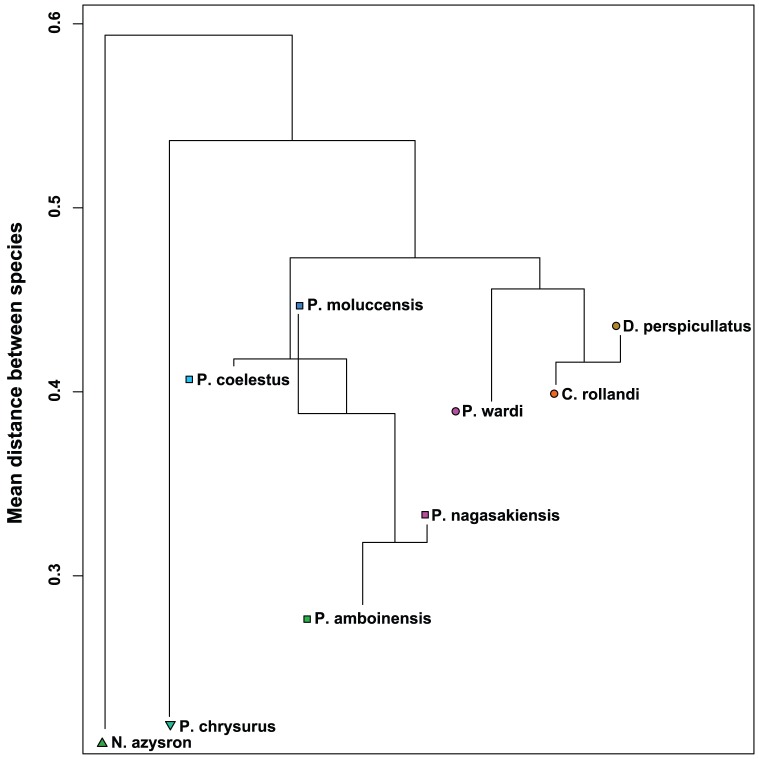
A dendrogram of species of fishes that behave similarly. Mean distances between species in multidimensional space plotted as a dendrogram to group similarly behaving species (small between-group distances). Termini indicate within-cluster dissimilarity for the species; horizontal lines indicate mean between-cluster dissimilarity. Termini that finish above the mean between-cluster line indicate species that are more heterogeneous than the combined cluster. Distance here refers to the positions of groups (group averages) within multidimensional behavioural space.

We next found that species behaviour differed significantly between groups (MRPP: A = 0.26, observed δ = 0.35, expected δ = 0.48, *p*<0.01). MRPP found four group clusterings, with *N. azysron's* behaviours showing the highest dissimilarity to all other species, and *P. chrysurus* also showing high dissimilarity from the remaining species (Fig. 2). The remaining species could be further broken into two behaviourally dissimilar clusters (cluster 1: *D. perspicillatus*, *C. rollandi* and *P. wardi*, cluster 2: *P. nagasakiensis*, *P. moluccensis*, *P. coelestis* and *P. amboinensis*), however, this dissimilarity was less marked than the dissimilarity of *N. azysron* and *P. chrysurus*. Finally, we investigated behavioural heterogeneity within species using PERMDISP2. We found significant differences in behavioural heterogeneity between species (F_9, 104_ = 5.9, *p*<0.01). *P. nagasakiensis* had the lowest behavioural heterogeneity (all individuals within the species behaved relatively similarly) and *P. moluccensis* had the highest (all individuals within the species behaved relatively dissimilarly; Table 4).

**Table 4:** Species' average dispersion (behavioural heterogeneity).

Species	Average distance from centroid	s.e.
*C. rollandi*	29.76	2.35
*D. perspiculatus*	29.64	1.96
*N. azysron*	22.36	2.02
*P. amboinensis*	17.94	3.90
*P. chrysurus*	15.50	1.93
*P. coelestus*	27.61	3.18
*P. moluccensis*	30.64	2.40
*P. nagasakiensis*	14.74	1.81
*P. wardi*	28.15	3.56

Dispersion is indicated by the average distance of each individual to each individual's species' centroid. Large values indicate high behavioural heterogeneity; low values indicate low behavioural heterogeneity.

## Discussion

The importance of interspecific comparative analyses for understanding the evolutionary basis of animal personality is recognised [9], [10], [11], however, few systems can accommodate these questions and adequate statistical tools for their investigation are lacking. At settlement, a plethora of coral reef fish species simultaneously recruit to the reef, providing an ideal system for interspecific comparative studies. Here, we investigated the usefulness of ecological community analysis tools (CVA, MRPP and PERMDISP2) for investigating interspecific variation in animal personality. First, we confirmed a behavioural syndrome across species, suggesting that animal personality is ecologically relevant in these species. We found that bolder individuals spent more time in risky areas of the patch reef, move more and had higher strike rates than shy individuals. We then used canonical variate analysis (CVA) to visually investigate species' differences in behaviour, and finally, we calculated behavioural dissimilarity between species as a measure of behavioural distinctiveness (how different are species behaviourally from one another?) and behavioural heterogeneity (how variable are species behaviourally from one another?) using MRPP and PERMDISP2, respectively. Using these techniques, we found significant variation between species in both the average behaviour species express and the variation in the behaviour they express.

These statistical techniques, although developed and widely used for the analysis of ecological communities, are quantitative, informative and easy to apply to analyses of animal behaviour. Previous studies that have compared between-species differences in behaviours have been ‘restricted’ to comparing one behaviour at a time, which are usually experimentally measured personality traits (such as exploration) [9], [11] or a ‘simplified’ behavioural score (such as time spent in open vs. vegetated water) [10]. Ecological community analysis tools can compare multiple behaviours and multiple species simultaneously and quantitatively, and we therefore encourage their use by future studies.

We found that the behaviours of *N. azysron* and *P. chrysurus* were significantly different to the behaviours of the seven other assayed species. Both expressed relatively low behavioural heterogeneity. *P. chrysurus* expressed, on average, the shyest responses to the boldness assay and spent relatively more time observing than the other species, while *N. azysron* spent relatively more time in the top third of the patch reef and at 10 cm from the patch reef than the other species (Fig. 1). These behaviours appear primarily responsible for the differentiation of the ‘out group’ species from the other clusters of species, which behaved more similarly to each other (Fig. 2). Although it was not our goal in this study to identify the reasons these fishes may behave differently to other fish species, determining the factors driving these behavioural tendencies may have implications for understanding the evolution of animal personality.

The observed differences in behaviour between *N. azysron* and *P. chrysurus* and the other species may be a result of their life history strategy [11], their external environment [39], [40] or a combination of both. For example, these two species appear the most distinct of the nine investigated species with respect to their aggregative tendencies at settlement. *N. azysron* tends to settle and live in schools [41], while newly settled *P. chrysurus* tend to avoid areas with adults (with whom they often have agonistic interactions) and be more solitary [42]; all other species measured tend to inhabit a wider variety of social environments [41], [42], [43]. Further, these two species may vary in their habitat choices. *N. azysron* tends to inhabit areas above the coral, feeding more in the water column [41], while *P. chrysurus* tends to live more amongst coral rubble [42], [44]; the other species vary predominantly between live corals, coral rubble and sand [41], [42], [43]. However, when considered together in the context of predation, which at this life history stage is extremely high and a strong selective force [17], [18], [19], [28], [45], it may be beneficial for the more solitary *P. chrysurus* to be shy and cautious of active predators which are abundant in these habitats (e.g. ambush predators such as the dottyback, *Pseudochromis fuscus*) [30] compared to the much bolder, schooling *N. azysron*, which sits on top of the reef and may benefit from a dilution effect against fast striking, long distance predators (e.g. the lizardfish, *Synodus englemani*) [31]. However, while this is an alluring and simple explanation, more targeted studies are required to disentangle the drivers of behavioural differences between these species.

We also found differences in average behavioural heterogeneity between the 9 species (Table 4). Previous work in other systems has found that selective forces, such as predation, can affect behavioural heterogeneity. For example, Edgell [46] showed that snails, *Litorina obtusata*, that had been exposed to predation by crabs, *Carcinus maenus*, for longer (100 *versus* 60 years) showed higher canalization in antipredator response, that is, lower behavioural flexibility, than snails exposed for shorter time periods [47], [48]. As predation is a strong selective force in coral reef systems at settlement [17], [19], it is tempting to suggest that this, perhaps coupled with variation of microhabitat choice (and therefore variation in commonly encountered predators) [30], [31] by these species [41], [42], [43], [44] may have resulted in the observed differences in behavioural heterogeneity between species. However, causal explanations of behavioural heterogeneity must be treated with caution, especially in samples taken from the wild. While canalising selection can decrease behavioural heterogeneity [46], other explanations, unrelated to selection *per se*, may equally generate heterogeneous/homogeneous behaviour in a species. Differences in the age, size or other latent variables of the sampled fish in our study may have artificially inflated the behavioural variation in some species. Further, differences in plastic responses to local environmental variation [49] may increase the heterogeneity of observed behaviours. Despite this, behavioural heterogeneity may be important in understanding species' differences in behaviour and personality and could be a useful tool in future studies.

Although we found evidence for a between-species behavioural syndrome, we found variable correlations between behavioural variables within species (see Supplementary Table S1). Interestingly, the two species with the lowest behavioural heterogeneity (Table 4) showed no evidence for a behavioural syndrome while three of the four species with highest behavioural heterogeneity (Table 4) showed the greatest evidence of a behavioural syndrome. Although the sample sizes within each species are relatively low, this finding raises two important points. First, if behavioural correlations or personality occur because of underlying neurological or hormonal ‘constraints’ or adaptive combinations of behaviours [50], [51], [52], [53], [54], then investigating where a ‘lack of personality’ occurs may be instructive in understanding the evolutionary requirements for these mechanisms [55]. Second, our results highlight the need to consider ‘situational strength’ during behavioural assays. Situational strength refers to how much an individual's behaviour is influenced by the test situation [56]. Strong situations may leave little variation between individual's behaviour whereas weaker situations may allow more inter-individual differences to show [57], [58]. In our study it is possible that species ‘experienced’ different situational strengths during the behavioural assays, which may result in differences in behavioural heterogeneity and thus correlations between behaviours. This is an important consideration for future comparative studies of animal personality.

Finally, comparisons of average behavioural characters, such as personality, across a variety of species are fundamental for investigations of how anthropogenic changes will affect marine communities. Coral reef systems are particularly vulnerable to anthropogenic changes such as climate change, and there is interest on how these processes affect fish behaviour and species' interactions [44], [59], [60], [61], [62]. These studies generally investigate the effects of different climate regimes on single species [59], [61], [62], however multiple-species comparisons are essential for investigating how these processes will affect communities [44] and drive the evolution of phenotypes, such as personality. Techniques such as those highlighted in this study may provide a useful ‘first-step’ for investigating how these processes affect communities, rather than the effects on single species, and affect the evolution of behavioural phenotypes.

In summary, intraspecific population and interspecific comparative analyses have the potential to help explain the nature and evolutionary significance of animal personalities. We suggest that using techniques such as ecological community analysis can help identify where similarities and differences in behaviour lie between species, after which more targeted studies can be performed to help gain and understanding of the drivers of animal personality differentiation between species.

## Supporting Information

Figure S1 **Biplots of A) principal components and B) canonical variates analysis of the behavioural data.** Individual fish plotted on A) principal components 1 and 2 and B canonical variates 1 and 2. CVA first analyses a matrix of variable values in much the same way as principal components analysis (PCA), however CVA maximises the variance explained between groups while PCA maximises the variation among individuals. The result is that species are more dispersed in the PCA (A), while species are more closely grouped with one another in the CVA (B). Further details can be found in: McCune, B., J.B. Grace, and D.L. Urban. 2002. ***Analysis of Ecological Communities.*** MjM Software Design. Each coloured line represents a behavioural variable; the labels are attached to the positive termini of each variable. Each symbol represents fish that were classified *a posteriori* to belong to a group of similarly behaving fish (see Fig. 2, main text); each colour represents a different fish species: *Chrysiptera rollandi* (orange circle), *Dischistodus perspicillatus* (brown circle), *Neopomacentrus azysron* (green upward pointing triangle), *Pomacentrus amboinensis* (light green square), *P. chrysurus* (aqua downward pointing triangle), *P. coelestis* (light blue square), *P. moluccensis* (blue square), *P. nagasakiensis* (purple square), *P. wardi* (pink circle). Large symbols in B indicate species centroids.
(EPS)

Table S1 **Species-level relationships between PC1 (boldness-activity behaviour) and PC2 (time budgets); individual boldness (startle response); and feeding success (strike rate).**
(DOC)
