# Supplementary material for: Taking a Comparative Approach: Analysing Personality as a Multivariate Behavioural Response across Species
Source: PLoS One. 2012 Jul 31;7(7):e42440. doi: 10.1371/journal.pone.0042440 (PMC3409165; doi:10.1371/journal.pone.0042440)
Supplement: Table S1 — Species-level relationships between PC1 (boldness-activity behaviour) and PC2 (time budgets); individual boldness (startle response); and feeding success (strike rate). (DOC) [file pone.0042440.s002.doc]

**Table S1**: Species-level relationships between PC1 (boldness-activity behaviour) and PC2 (time budgets); individual boldness (startle response); and feeding success (strike rate).

| Species | n | Response | Predictor | Estimate | Standard Error | *t* value | *p* value |
| --- | --- | --- | --- | --- | --- | --- | --- |
| *C. rollandi* | 12 | Strike rate | Intercept | 4.69 | 0.11 | 43.84 | <0.001 |
|  | 12 |  | PC1 | -0.09 | 0.03 | -2.92 | **0.02** |
|  | 12 |  | PC2 | 0.01 | 0.07 | 0.16 | 0.88 |
|  | 12 | Startle response | Intercept | 2.72 | 0.29 | 9.55 | <0.001 |
|  | 12 |  | PC1 | -0.06 | 0.09 | -0.62 | 0.55 |
|  | 12 |  | PC2 | 0.10 | 0.18 | 0.55 | 0.59 |
|  | 12 | Startle response | Intercept | 2.02 | 0.65 | 3.09 | 0.01 |
|  | 12 |  | Strike rate | 0.01 | 0.01 | 0.88 | 0.40 |
| *D. perspicullatus* | 10 | Strike rate | Intercept | 4.05 | 0.08 | 52.31 | <0.001 |
|  | 10 |  | PC1 | -0.09 | 0.04 | -2.26 | **0.06** |
|  | 10 |  | PC2 | -0.02 | 0.04 | -0.66 | 0.53 |
|  | 10 | Startle response | Intercept | 2.25 | 0.29 | 7.64 | <0.001 |
|  | 10 |  | PC1 | -0.41 | 0.15 | -2.75 | **0.03** |
|  | 10 |  | PC2 | -0.10 | 0.14 | -0.73 | 0.49 |
|  | 10 | Startle response | Intercept | -0.89 | 0.97 | -0.91 | 0.39 |
|  | 10 |  | Strike rate | 0.05 | 0.02 | 2.93 | **0.02** |
| *N. azysron* | 10 | Strike rate | Intercept | 4.26 | 0.38 | 11.23 | <0.001 |
|  | 10 |  | PC1 | -0.03 | 0.12 | -0.26 | 0.80 |
|  | 10 |  | PC2 | -0.10 | 0.04 | -2.44 | **0.04** |
|  | 10 | Startle response | Intercept | 3.64 | 2.54 | 1.43 | 0.19 |
|  | 10 |  | PC1 | 0.19 | 0.81 | 0.24 | 0.82 |
|  | 10 |  | PC2 | -0.28 | 0.26 | -1.07 | 0.32 |
|  | 10 | Startle response | Intercept | -0.53 | 1.38 | -0.39 | 0.71 |
|  | 10 |  | Strike rate | 0.05 | 0.02 | 2.30 | **0.05** |
| *P. amboinensis* | 10 | Strike rate | Intercept | 4.27 | 0.05 | 80.84 | <0.001 |
|  | 10 |  | PC1 | -0.13 | 0.05 | -2.49 | **0.04** |
|  | 10 |  | PC2 | -0.15 | 0.07 | -2.15 | 0.07 |
|  | 10 | Startle response | Intercept | 1.95 | 0.17 | 11.18 | <0.001 |
|  | 10 |  | PC1 | -0.15 | 0.15 | -0.97 | 0.37 |
|  | 10 |  | PC2 | 0.21 | 0.25 | 0.82 | 0.44 |
|  | 10 | Startle response | Intercept | 1.85 | 0.59 | 3.17 | 0.01 |
|  | 10 |  | Strike rate | <0.001 | 0.01 | 0.26 | 0.80 |
| *P. chrysurus* | 12 | Strike rate | Intercept | 4.12 | 0.16 | 25.32 | <0.001 |
|  | 12 |  | PC1 | 0.01 | 0.10 | 0.05 | 0.96 |
|  | 12 |  | PC2 | -0.42 | 0.27 | -1.58 | 0.15 |
|  | 12 | Startle response | Intercept | 0.98 | 0.42 | 2.32 | 0.05 |
|  | 12 |  | PC1 | -0.28 | 0.24 | -1.17 | 0.27 |
|  | 12 |  | PC2 | 0.04 | 0.57 | 0.07 | 0.95 |
|  | 12 | Startle response | Intercept | -0.14 | 0.63 | -0.22 | 0.83 |
|  | 12 |  | Strike rate | 0.01 | 0.01 | 0.79 | 0.45 |
| *P. coelestus* | 15 | Strike rate | Intercept | 4.60 | 0.14 | 32.42 | <0.001 |
|  | 15 |  | PC1 | -0.10 | 0.06 | -1.70 | 0.11 |
|  | 15 |  | PC2 | -0.16 | 0.07 | -2.32 | **0.04** |
|  | 15 | Startle response | Intercept | 2.58 | 0.27 | 9.66 | <0.001 |
|  | 15 |  | PC1 | -0.07 | 0.12 | -0.58 | 0.57 |
|  | 15 |  | PC2 | 0.08 | 0.14 | 0.58 | 0.57 |
|  | 15 | Startle response | Intercept | 3.21 | 0.57 | 5.63 | <0.001 |
|  | 15 |  | Strike rate | <0.001 | <0.001 | -1.12 | 0.28 |
| *P. moluccensis* | 13 | Strike rate | Intercept | 3.92 | 0.09 | 42.47 | <0.001 |
|  | 13 |  | PC1 | -0.07 | 0.05 | -1.45 | 0.18 |
|  | 13 |  | PC2 | -0.19 | 0.06 | -3.10 | **0.01** |
|  | 13 | Startle response | Intercept | 2.29 | 0.08 | 28.23 | <0.001 |
|  | 13 |  | PC1 | -0.21 | 0.05 | -4.18 | **<0.001** |
|  | 13 |  | PC2 | -0.22 | 0.07 | -3.02 | **0.01** |
|  | 13 | Startle response | Intercept | 1.20 | 0.34 | 3.56 | <0.001 |
|  | 13 |  | Strike rate | 0.02 | 0.01 | 3.81 | **<0.001** |
| *P. nagasakiensis* | 10 | Strike rate | Intercept | 4.43 | 0.04 | 107.89 | <0.001 |
|  | 10 |  | PC1 | 0.03 | 0.04 | 0.58 | 0.58 |
|  | 10 |  | PC2 | -0.05 | 0.04 | -1.29 | 0.24 |
|  | 10 | Startle response | Intercept | 2.48 | 0.25 | 10.06 | <0.001 |
|  | 10 |  | PC1 | -0.20 | 0.27 | -0.77 | 0.47 |
|  | 10 |  | PC2 | 0.07 | 0.26 | 0.26 | 0.81 |
|  | 10 | Startle response | Intercept | 5.66 | 1.67 | 3.39 | 0.01 |
|  | 10 |  | Strike rate | -0.04 | 0.02 | -1.90 | 0.09 |
| *P. wardi* | 12 | Strike rate | Intercept | 4.23 | 0.06 | 70.52 | <0.001 |
|  | 12 |  | PC1 | -0.05 | 0.02 | -2.04 | **0.07** |
|  | 12 |  | PC2 | -0.33 | 0.05 | -6.13 | **<0.001** |
|  | 12 | Startle response | Intercept | 1.77 | 0.23 | 7.69 | <0.001 |
|  | 12 |  | PC1 | 0.05 | 0.11 | 0.43 | 0.68 |
|  | 12 |  | PC2 | -0.59 | 0.22 | -2.72 | **0.02** |
|  | 12 | Startle response | Intercept | 0.54 | 0.50 | 1.08 | 0.30 |
|  | 12 |  | Strike rate | 0.02 | 0.01 | 2.49 | **0.03** |
